# Investigation of bioaccessibility of Cu, Fe, Mn, and Zn in market vegetables in the colon using PBET combined with SHIME

**DOI:** 10.1038/s41598-017-17901-1

**Published:** 2017-12-14

**Authors:** Naiyi Yin, Xiaolin Cai, Xiaochen Chen, Huili Du, Jiayan Xu, Lihong Wang, Guoxin Sun, Yanshan Cui

**Affiliations:** 1 College of Resources and Environment, University of Chinese Academy of Sciences, Beijing, 101408 People’s Republic of China; 2 College of Environment and Resources, Fuzhou University, Fuzhou, Fujian, 350116 People’s Republic of China; 3 Research Center for Eco-Environmental Sciences, Chinese Academy of Sciences, Beijing, 100085 People’s Republic of China; 4 Shandong Analysis and Test Center, Shandong Academy of Sciences, Jinan, Shandong 250014 People’s Republic of China

## Abstract

The *in vitro* bioaccessibility of trace metals associated with oral ingestion of market vegetables (lettuce, pak choi, cole, and leaf lettuce) of Beijing, China was studied. The physiologically based extraction test (PBET) combined with the Simulator of Human Intestinal Microbial Ecosystem (SHIME) was applied to simulate stomach, small intestine, and colon of human. In the gastro-intestinal phases, the bioaccessibility of Cu, Fe, Mn, and Zn varied within 5.7–75.5%, 17.3–50.4%, 13.3–49.1%, and 19.9–63.7%, respectively. There was no significant difference in the metal bioaccessibility between the gastric and small intestinal phases, except for higher Cu bioaccessibility in the small intestine. Besides, the bioaccessibility of the four trace metals in the colon phase was first ever reported. A significant decline in Cu bioaccessibility (1.8–63.7%) and slight increases in the bioaccessibility of Fe (16.7–56.4%), Mn (21.2–71.6%), and Zn (15.7–69.7%) were revealed, which could mainly be attributed to the effect of colon microbiota. In addition, the estimated daily intakes (EDIs) of Cu, Fe, Mn, and Zn were worked out to be 0.7, 8.8, 2.7, and 4.5 μg kg^−1^ body weight d^−1^, based on which the potential influences of these trace metals in vegetables on the health of the local consumers was demonstrated.

## Introduction

Food nutrition has always been receiving broad attention. The daily oral ingestion of food is the major way, through which more than 90% of nutrients/contaminants enters human body^1^. As one of the major foods consumed in human diet, vegetables are rich in carbohydrates, vitamins, and minerals. Several trace metals as components of enzymes are essential for life, such as copper (Cu), iron (Fe), manganese (Mn), and zinc (Zn)^2^. However, the over-accumulation of the heavy metals in vegetables, as well as the excessive intake of these essential elements, may induce negative effects on human health^3^.

Bioaccessibility (*in vitro* studies) is defined as the fraction of metals that is soluble in the gastrointestinal environment of human and available for absorption, whereas bioavailability (*in vivo* studies) is determined by the fraction of metals that is eventually absorbed into the systemic circulation. Studies on the *in vivo*-*in vitro* correlation demonstrated the validity of *in vitro* methods for predicting relative bioavailability (RBA) of metals in foods and soils, such as arsenic and mercury^4–6^. The *in vitro* simulation of the human digestive tract including stomach and small intestine has been realized, and the physiologically based extraction test (PBET) is one of the most commonly applied methods in this field^7^. In an oral ingestion scenario, the *in vitro* gastrointestinal methods were applied to estimate the bioaccessibility of heavy metals in various food sources, such as rice, vegetables, and seafood^8–10^. Besides, some factors affecting the bioaccessible metals in the gastric and small intestinal phases were studied, including vegetable species, cooking, and the difference in fresh and dry samples^11–13^. By far, however, there has been limited knowledge about the bioaccessibility of metals in market vegetables.

As is known to all, small intestine is a key part of the digestive system of human, where the absorption of nutrients and minerals mainly takes place. Dissolved metals are readily absorbed in the small intestine^14^, especially for the case of a soluble matrix. However, it was usually ignored that soil/food-bound metals with different digestion scenarios consequentially get into the colon, where abundant microbial community inhabits. Simulator of Human Intestinal Microbial Ecosystem (SHIME), a dynamic human gastrointestinal simulator, achieves *in vitro* culture of the colon microbial community of human origin. The explorations with SHIME thoroughly revealed that human gut microbiota had high metabolic potency toward xenobiotics^15,16^. Besides, the importance of gut microbiota on the metabolism of metal(loid)s was demonstrated, as they significantly affected the bioaccessibility and biotransformation of the metal(loid)s in foods and soils, such as arsenic and selenium^10,17,18^. Considering the significance of presystemic metabolism in the human body, it was deemed indispensable to investigate the effects of gut microbiota on metal bioaccessibility associated with oral food ingestion. The previous *in vitro* bioaccessibility studies mainly focused on simulating the gastric and small intestinal phases. Unfortunately, regarding market vegetables there have been no data about the bioaccessibility of Cu, Fe, Mn, and Zn in the colon phase.

In this study, the PBET method (gastric and small intestinal phases) combined with SHIME (colon phase) was used to thoroughly evaluate the oral bioaccessibility of Cu, Fe, Mn, and Zn in the market vegetables of the Beijing city proper, China. Accordingly, we worked out the daily intakes and the corresponding contributions to the local consumers. This investigation is valuable for more accurate assessment of the potential health impact of these essential trace metals.

## Materials and Methods

### Ethical approval and informed consent

All experiments were carried out in accordance with the relevant guidelines and regulations of all the participants’ institutions including Chinese Academy of Sciences, Fuzhou University, and Shandong Academy of Sciences, and were hence approved by the institutional committees. Informed consent was obtained from all the participants prior to the commencement of the study.

### Sample collection, preparation, and analysis

Four most common leafy vegetables were randomly purchased from four major vegetable markets (M1, M2, M3, and M4) located in different districts of Beijing, China, including lettuce (*Lactvca saiva* L.), pak choi (*Brassica chinensis* L.), cole (*Brassica campestris* L.), and leaf lettuce (*Lactuca sativa* var *longifolia* f. Lam). Fresh vegetables were thoroughly washed with deionized water to remove visible dirt and patted dry for the following study. Moisture content was measured by oven-drying at 70 °C for 48 h. After microwave digestion (MARS 6, CEM, U.S.) using concentrated HNO_3_, total metals (Cu, Fe, Mn, and Zn) in fresh vegetables were determined by inductively coupled plasma-optical emission spectroscopy (ICP-OES, PerkinElmer Optima 7300 V, U.S.) or inductively coupled plasma mass spectrometry (ICP-MS, Agilent 7500a, U.S.). In the digestion process, blank samples and reference material (GBW10015, Institute of Geophysical & Geochemical Exploration, China) were included for the internal quality assurance/quality control (QA/QC), and the recoveries were 93.5–102.2% (*n* = 3).

### Dynamic SHIME

The SHIME consisted of five compartments simulating the stomach, small intestine, ascending colon, transverse colon and descending colon. According to Van de Wiele *et al*.^19^ and Yin *et al*.^18^, fresh fecal microorganisms were obtained from one 28-year-old Chinese male volunteer with no history of antibiotic treatment within the six months before this study. Briefly, the fresh fecal microorganisms were inoculated in the three colon compartments. Feed solution specified by Yin *et al*.^20^ was refreshed three times per day to provide digested nutrition for the colon microorganisms. The temperature (37 °C) and pH (5.6–5.9, 6.15–6.4, and 6.7–6.9 in the ascending colon, transverse colon and descending colon, respectively) were automatically maintained. The SHIME reactor was continuously stirred and kept under anaerobic environment by regular flushing with nitrogen gas. After about four weeks of adaptation, stable microbial communities were obtained in the colon compartments. The fermentation activity (production of short chain fatty acid and ammonium) and community composition of the distal colon microorganisms were consistent with that of previous SHIME runs and an *in vivo* situation^18,21^.

### *In vitro* digestion

The oral bioaccessibility of Cu, Fe, Mn, and Zn in fresh vegetables was investigated using PBET (gastric and small intestinal phases) combined with SHIME (colon phase), with the detailed procedures described by Fu *et al*.^12^ and Yin *et al*.^22^. Briefly, the fresh vegetable sample (3 g) in triplicate was added to a polypropylene conical centrifuge tube (50 mL) with the digest (30 mL) at a solid/solution (s/s) ratio of 1:10 in the gastric and small intestinal phases. Following the small intestinal phase, the digest was transferred into an anaerobic serum bottle (100 mL) with colon solution (30 mL) from the descending colon compartment of the dynamic SHIME system at a ratio (s/s) of 1:20. Subsequently, the bottle was capped with a butyl rubber stopper and immediately flushed with nitrogen gas for 20–30 min to obtain anaerobic condition. The digest was then shaken at 150 rpm for 18 h. The entire *in vitro* digestion process was maintained at 37 °C. Samples taken at the end of each phase were centrifuged at 4000 g for 20 min. The supernatant was filtered (0.22 μm, Millipore, U.S.) and stored at −20 °C prior to ICP-OES/MS analysis.

### Statistical analysis

Analysis of variance and paired-samples T test were performed to determine the significance of differences regarding the bioaccessibility of Cu, Fe, Mn, and Zn in the fresh market vegetables (at the 0.05 level) using SPSS software (version 20.0, IBM, U.S.). Spearman’s correlation analysis between the metal bioaccessibility, bioaccessible concentrations and total concentrations was performed by SPSS as well.

### Data availability statements

All data generated or analysed during this study are included in this published article.

## Results and Discussion

### Total concentrations of metals in the vegetables

The total concentrations of Cu, Fe, Mn, and Zn in the fresh vegetables were 0.4–5.5, 7.6–26.0, 1.9–17.3, and 5.6–15.3 mg kg^−1^, respectively (Table 1), which were acceptable according to the Chinese thresholds^23^. For all the vegetables from all the markets, Fe showed a highest average concentration and Cu showed the lowest average concentration. Moisture content of all vegetables exceeded 95%.

**Table 1:** Total concentrations of Cu, Fe, Mn, and Zn in the fresh market vegetables (*n* = 3).

Samples		Cu	Fe	Mn	Zn
M1	LT	1.1 ± 0.3	14.7 ± 0.1	3.9 ± 0.1	8.9 ± 0.2
	PC	0.8 ± 0.0	18.8 ± 1.1	4.4 ± 0.2	6.5 ± 0.2
	CL	1.0 ± 0.1	18.5 ± 0.5	5.3 ± 0.4	7.9 ± 1.0
	LL	1.5 ± 0.1	15.2 ± 1.7	3.1 ± 0.0	6.9 ± 1.4
M2	LT	0.7 ± 0.0	16.7 ± 1.0	4.8 ± 0.1	5.8 ± 1.0
	PC	0.7 ± 0.1	25.9 ± 1.6	4.5 ± 0.7	6.3 ± 1.2
	CL	1.3 ± 0.2	24.7 ± 1.7	4.7 ± 0.5	12.4 ± 1.5
	LL	5.5 ± 0.8	14.5 ± 0.1	4.6 ± 1.2	5.6 ± 0.5
M3	LT	0.9 ± 0.1	11.8 ± 0.8	3.9 ± 0.5	8.4 ± 1.1
	PC	0.8 ± 0.0	26.0 ± 2.9	7.4 ± 0.9	8.1 ± 1.4
	CL	1.6 ± 0.6	18.6 ± 3.4	17.3 ± 0.2	12.0 ± 2.1
	LL	0.9 ± 0.1	17.9 ± 1.4	4.8 ± 0.1	5.8 ± 0.4
M4	LT	0.4 ± 0.0	7.6 ± 1.1	1.9 ± 0.3	5.8 ± 0.7
	PC	0.9 ± 0.0	19.0 ± 2.3	4.3 ± 0.2	15.3 ± 1.9
	CL	1.2 ± 0.2	14.1 ± 1.2	4.7 ± 0.3	8.8 ± 0.7
	LL	1.3 ± 0.1	16.3 ± 1.5	4.0 ± 0.1	6.5 ± 0.0

M1, M2, M3, and M4: markets 1–4.

LT: lettuce; PC: pak choi; CL: cole; LL: leaf lettuce.

Values are mean ± SD (mg/kg).

### Bioaccessibility of metals in the vegetables

**Figure 1:**
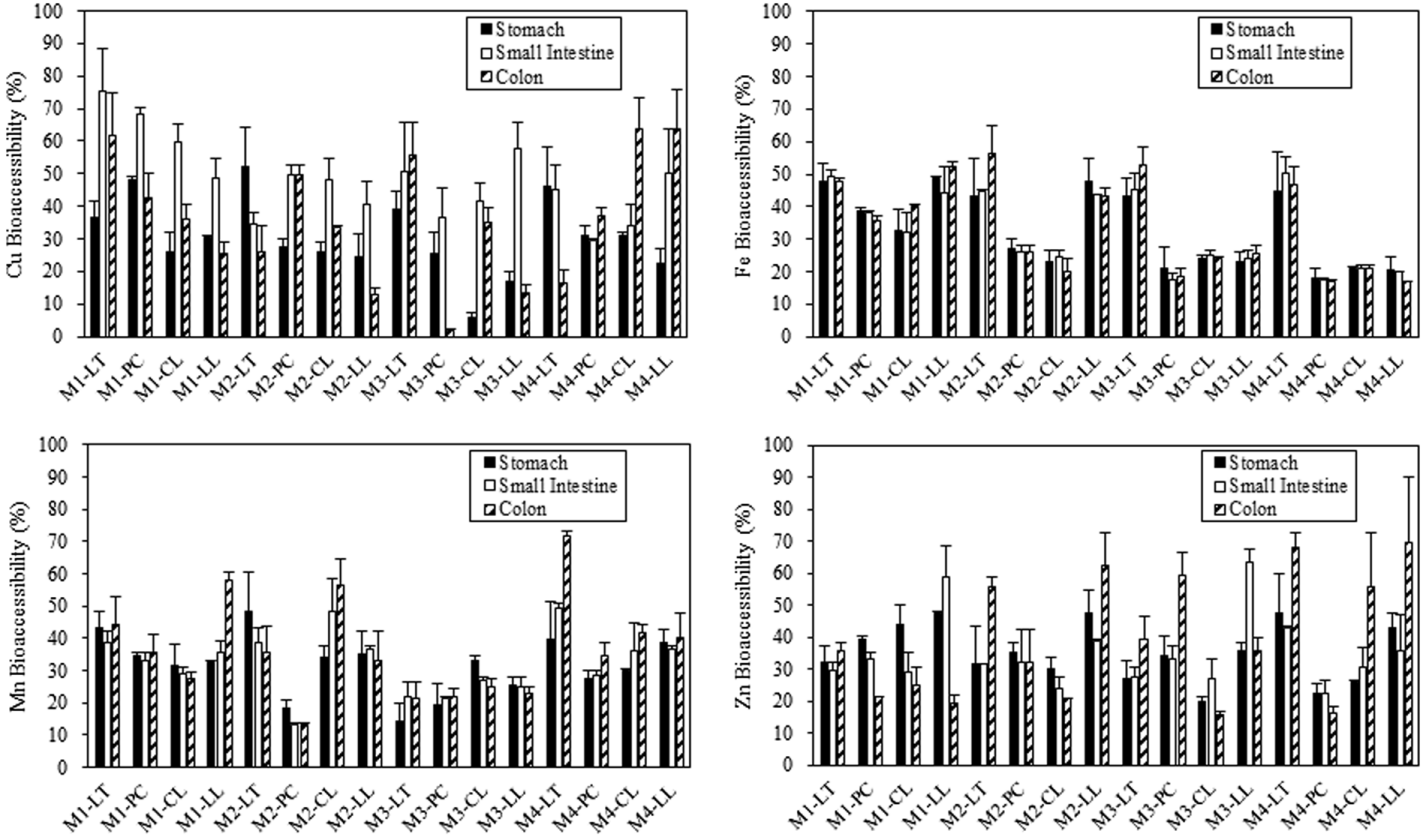
Bioaccessibility (%) of Cu, Fe, Mn, and Zn in the fresh market vegetables in the gastric, small intestinal, and colon phases. Data are presented as mean ± SD (*n* = 3). (M1, M2, M3, and M4: markets 1–4. LT: lettuce; PC: pak choi; CL: cole; LL: leaf lettuce.)

The bioaccessible concentrations and bioaccessibility of Cu, Fe, Mn, and Zn in fresh vegetables are shown in Table 2 and Fig. 1. In the gastro-intestinal phases, the bioaccessible concentrations and bioaccessibility were 0.1–2.2 mg kg^−1^ and 5.7–75.5% for Cu, 3.0–7.5 mg kg^−1^ and 17.3–50.4% for Fe, 0.6–5.8 mg kg^−1^ and 13.3–49.1% for Mn, and 1.8–4.1 mg kg^−1^ and 19.9–63.7% for Zn. From the gastric to the small intestinal phase, Cu bioaccessibility significantly increased for most of the vegetable samples (paired-samples T test, *p* < 0.05), which on average doubled. For the bioaccessibility of Fe, Mn, and Zn, no significant difference was observed between the gastric and small intestinal phases (paired-samples T test, *p* > 0.05). With the progress of digestion, more metals in the vegetables could be dissolved into the digestive juice. However, due to the dramatic increase of pH and the introduction of more enzymes in the small intestine, a series of reactions such as complexation, adsorption and precipitation could happen to the metal ions. As a result, the difference of bioaccessibility between the gastric and small intestinal phases turned out to be non-significant. As for the exceptional bioaccessibility increase of Cu, it could be partially attributed to the good solubility of Cu-phytate complexes in the intestinal lumen^24,25^. Further studies are recommended to clarify the exact mechanisms. In the limited amount of investigations regarding metal bioaccessibility in the vegetables obtained from markets or grown in contaminated soils^9,13,26,27^, the reported data were basically comparable to ours. In the gastric and small intestinal phases, the average bioaccessibility was 16% and 18% for Cu, 43% and 25% for Zn in some leafy vegetables^27^. Cu bioaccessibility varied within 32% and 15% in lettuce, and 38% and 26% in leaf lettuce^9^. da Silva *et al*.^26^ found Fe and Zn bioaccessibility were 23.6% and 39.2% in lettuce, and 52.9% and 36.8% in cole.

**Table 2:** Bioaccessible concentrations of Cu, Fe, Mn, and Zn in the fresh market vegetables in the gastric, small intestinal, and colon phases (*n* = 3).

Samples		Cu	Fe	Mn	Zn
M1	LT-G	0.4 ± 0.1	7.1 ± 0.6	1.7 ± 0.1	2.8 ± 0.1
	LT-I	0.8 ± 0.1	7.3 ± 0.2	1.5 ± 0.1	2.6 ± 0.2
	LT-C	0.7 ± 0.2	7.0 ± 0.1	1.7 ± 0.3	3.2 ± 0.2
	PC-G	0.4 ± 0.0	7.2 ± 0.1	1.5 ± 0.2	2.5 ± 0.1
	PC-I	0.5 ± 0.0	7.2 ± 0.1	1.5 ± 0.1	2.2 ± 0.1
	PC-C	0.3 ± 0.1	6.7 ± 0.3	1.6 ± 0.3	1.4 ± 0.1
	CL-G	0.3 ± 0.1	6.0 ± 0.6	1.7 ± 0.3	3.4 ± 0.9
	CL-I	0.6 ± 0.1	5.9 ± 1.2	1.6 ± 0.1	2.3 ± 0.5
	CL-C	0.4 ± 0.1	7.4 ± 0.1	1.5 ± 0.1	2.0 ± 0.4
	LL-G	0.5 ± 0.0	7.4 ± 0.2	1.0 ± 0.1	3.3 ± 0.8
	LL-I	0.7 ± 0.1	6.8 ± 1.2	1.1 ± 0.1	4.1 ± 0.7
	LL-C	0.4 ± 0.1	8.0 ± 0.2	1.8 ± 0.1	1.3 ± 0.2
M2	LT-G	0.4 ± 0.1	7.2 ± 0.6	2.3 ± 0.4	1.8 ± 0.4
	LT-I	0.3 ± 0.0	7.5 ± 0.1	1.9 ± 0.2	1.8 ± 0.1
	LT-C	0.2 ± 0.1	9.4 ± 1.4	1.7 ± 0.4	3.2 ± 0.2
	PC-G	0.2 ± 0.0	7.1 ± 0.4	0.8 ± 0.1	2.2 ± 0.1
	PC-I	0.4 ± 0.0	6.8 ± 0.4	0.6 ± 0.1	2.0 ± 0.7
	PC-C	0.3 ± 0.0	6.2 ± 1.0	1.0 ± 0.1	3.4 ± 0.1
	CL-G	0.3 ± 0.1	5.7 ± 0.7	1.6 ± 0.3	3.8 ± 0.7
	CL-I	0.6 ± 0.1	6.1 ± 0.5	2.3 ± 0.5	3.0 ± 0.4
	CL-C	0.4 ± 0.0	5.0 ± 1.0	2.6 ± 0.4	2.6 ± 0.1
	LL-G	1.3 ± 0.4	6.9 ± 0.5	1.6 ± 0.4	2.7 ± 0.1
	LL-I	2.2 ± 0.4	6.3 ± 0.1	1.7 ± 0.1	2.2 ± 0.1
	LL-C	0.7 ± 0.1	6.2 ± 0.4	1.5 ± 0.4	3.5 ± 0.6
M3	LT-G	0.4 ± 0.1	5.1 ± 0.9	0.6 ± 0.1	2.3 ± 0.1
	LT-I	0.5 ± 0.1	5.4 ± 0.6	0.8 ± 0.2	2.3 ± 0.3
	LT-C	0.5 ± 0.1	6.2 ± 0.7	0.8 ± 0.2	3.3 ± 0.6
	PC-G	0.2 ± 0.1	5.4 ± 0.3	1.4 ± 0.1	2.8 ± 0.4
	PC-I	0.3 ± 0.1	4.5 ± 0.6	1.6 ± 0.1	2.7 ± 0.3
	PC-C	0.0 ± 0.0	4.8 ± 0.7	1.6 ± 0.2	4.8 ± 0.6
	CL-G	0.1 ± 0.0	4.4 ± 0.7	5.8 ± 0.2	2.4 ± 0.2
	CL-I	0.7 ± 0.1	4.7 ± 0.3	4.7 ± 0.2	3.2 ± 0.7
	CL-C	0.6 ± 0.1	4.5 ± 0.1	4.3 ± 0.4	1.9 ± 0.1
	LL-G	0.2 ± 0.0	4.1 ± 0.9	1.2 ± 0.1	2.1 ± 0.4
	LL-I	0.5 ± 0.1	4.3 ± 0.4	1.2 ± 0.2	3.7 ± 0.2
	LL-C	0.1 ± 0.0	4.6 ± 0.5	1.1 ± 0.1	2.1 ± 0.2
M4	LT-G	0.2 ± 0.1	3.4 ± 0.7	0.8 ± 0.1	2.8 ± 0.6
	LT-I	0.2 ± 0.0	3.8 ± 0.4	1.0 ± 0.1	2.5 ± 0.1
	LT-C	0.1 ± 0.0	3.5 ± 0.4	1.4 ± 0.1	3.9 ± 0.3
	PC-G	0.3 ± 0.0	3.4 ± 0.1	1.2 ± 0.1	3.4 ± 0.7
	PC-I	0.3 ± 0.0	3.3 ± 0.1	1.2 ± 0.1	3.4 ± 0.6
	PC-C	0.4 ± 0.0	3.2 ± 0.1	1.5 ± 0.2	2.5 ± 0.3
	CL-G	0.4 ± 0.0	3.0 ± 0.1	1.4 ± 0.1	2.3 ± 0.5
	CL-I	0.4 ± 0.1	3.0 ± 0.2	1.7 ± 0.4	2.7 ± 0.5
	CL-C	0.7 ± 0.1	3.0 ± 0.1	1.9 ± 0.1	4.9 ± 1.5
	LL-G	0.3 ± 0.1	3.3 ± 0.1	1.5 ± 0.1	2.8 ± 0.1
	LL-I	0.7 ± 0.2	3.3 ± 0.1	1.5 ± 0.1	2.3 ± 0.8
	LL-C	0.8 ± 0.2	2.7 ± 0.1	1.6 ± 0.3	4.5 ± 1.3

M1, M2, M3, and M4: markets 1–4.

LT: lettuce; PC: pak choi; CL: cole; LL: leaf lettuce.

G, I, and C represent the gastric, small intestinal, and colon phase, respectively.

Values are mean ± SD (mg/kg).

Following the transition from the small intestinal to the colon phase, Cu bioaccessibility declined for most of the vegetable samples, whereas the bioaccessibility of Fe, Mn, and Zn showed an increase (paired-samples T test, *p* < 0.05). The bioaccessible concentrations and bioaccessibility of Cu, Fe, Mn, and Zn was 0.02–0.8 mg kg^−1^ and 1.8–63.7%, 2.7–9.4 mg kg^−1^ and 16.7–56.4%, 0.8–4.3 mg kg^−1^ and 21.2–71.6%, and 1.3–4.9 mg kg^−1^ and 15.7–69.7%, respectively. To our knowledge, this was the first ever report of the bioaccessibility of Cu, Fe, Mn, and Zn in the market vegetables in the colon, which suggested that the previous *in vitro* studies without simulating the colon phase could have overestimated the bioaccessibility and bioavailability of Cu while underestimated those of Fe, Mn, and Zn. Under the anaerobic environment of the colon, human gut microbiota could induce reductive dissolution of Fe and Mn^22^, although the secondary Fe/Mn (hydr)oxides formation could inhibit their dissolution^18,28^. Besides, these trace metals present as organometallic salts in vegetables could be nutritional supplementation to the gut microbiota, resulting in their further release in the colon. Some organic acids in the colon digests, particularly for the abundant short chain fatty acids, could lead to Cu adsorption and hence reduced Cu dissolution^29^. Also, sulfide production by sulfate-reducing bacteria in the human gut^30^ could induce the precipitation of Cu and Zn sulfides and further reduced their dissolution in the neutral condition.

There was also significant difference in the metal bioaccessibility between markets and vegetable species (Univariate analysis of variance, *p* < 0.05). The bioavailability and bioaccessibility of trace metals (e.g., Cu, Fe, Mn, and Zn) could depend on their speciation in vegetables, which is also an important factor determining the nutritional quality. Some techniques have been used for studying metal speciation in foods and plants, such as size exclusion, ion chromatography, and X-ray absorption spectroscopy^31,32^. Trace metals are primarily complexed with organics in various molecular weights^33,34^, although some inorganic metal salts and soluble organometallics are readily to be released into the digestive system. In addition, chemical inhibition could lead to a significant reduction in metal dissolution, such as *S*-amino acids and histidine for Cu^35^, and nucleic acids and Ca for Zn^36^. The total concentrations in the vegetables might also play a role in affecting the digestion of metals (Table 3)^37^. Therefore, the change of metal bioaccessibility could be the result of concurrent processes.

**Table 3:** Correlation matrix between the bioaccessibility, bioaccessible concentrations and total concentrations of Cu, Fe, Mn, and Zn in the fresh market vegetables (n = 48).

	TCu	TFe	TMn	TZn
Cu-BA	−0.098	−0.171	−0.254*	0.082
Fe-BA	−0.087	−0.566**	−0.404**	−0.388**
Mn-BA	0.201*	−0.363**	−0.312**	−0.043
Zn-BA	−0.186	−0.301**	−0.175	−0.609**
Cu-BAC	0.611**	−0.268**	−0.124	0.131
Fe-BAC	−0.062	0.094	−0.006	−0.226*
Mn-BAC	0.453**	0.158	0.481**	0.234*
Zn-BAC	0.114	−0.031	−0.102	0.178

T: total concentrations of the trace metals in the vegetables.

BA: bioaccessibility.

BAC: bioaccessible concentrations.

*p < 0.05; **p < 0.01.

### Estimated daily intakes of metals from the vegetables

In order to evaluate the potential health impact on the local consumers of Beijing city proper via consumption of vegetables, we calculated the estimated daily intakes (EDIs) of the four trace metals, based on their bioaccessible concentrations, the consumption of 100 g of vegetables per day, and the average body weight of 62 kg for adults^27^. Seen in Table 4, the average EDIs of Cu, Fe, Mn, and Zn were 0.7, 8.8, 2.7, and 4.5 μg kg^−1^ d^−1^. As an estimate of the daily oral exposure of human population to metals, the average contributions of EDI to oral reference dose (*R*_*f*_ *D*)^38^ were 1.8%, 1.8%, and 1.5% for Cu, Mn, and Zn, respectively (Table 4). Hu *et al*.^27^ reported the average EDIs of Cu and Zn were 1.6 and 6.3 μg kg^−1^ d^−1^ from the market vegetables in Hong Kong. Pan *et al*.^9^ found higher EDI of Cu (2.8 μg kg^−1^ d^−1^) from the vegetables grown in contaminated soils.

**Table 4:** Estimated daily intakes (EDIs) of the bioaccessible metals in the market vegetables.

	EDI	*R*_*f*_ *D* ^a^	Average contribution rate of EDI to *R*_*f*_ *D*	DRI^b^	Average contribution rate of vegetable consumption to meet DRI
	μg kg^−1^ body weight d^−1^		%	mg d^−1^	%
Cu	0.02–3.6 (0.7)	40	1.8	0.8	5.7
Fe	4.4–15.2 (8.8)			12.0	4.5
Mn	0.9–9.3 (2.7)	150	1.8	4.5	3.7
Zn	2.2–7.9 (4.5)	300	1.5	12.5	2.3

^a^ *R*_*f*_ *D* obtained from the Integrated Risk Information Systems Database by USEPA.

^b^DRI as published by the Chinese Nutrition Society.

Regarding the daily reference intake (DRI) for nutrition recommendations^39^, the average contributions of consuming 100 g of a fresh vegetable to meet DRI were 5.7% for Cu, 4.5% for Fe, 3.7% for Mn, and 2.3% for Zn, respectively. This study suggested the leafy vegetables could also be an indispensable contributor of essential trace metals. Our investigation provides valuable reference for accurate assessment of the nutritional value of vegetables.

## Conclusions

The present study showed the variation of oral bioaccessibility of Cu, Fe, Mn, and Zn in different market vegetables of Beijing, China. Particularly, their bioaccessibility data in the colon were first reported. In the colon phase, Cu bioaccessibility significantly declined, while the bioaccessibility of Fe, Mn, and Zn slightly increased, which could mainly be attributed to the gut microbiota inhabiting the colon. Based on the estimated daily intakes, potential health effect of these trace metals in market vegetables on local consumers was further demonstrated.
